# Cell immobilization for enhanced milk clotting enzyme production from *Bacillus amyloliquefacien* and cheese quality

**DOI:** 10.1186/s12934-024-02521-y

**Published:** 2024-10-18

**Authors:** Eman A. Karam, Mohamed E. Hassan, Nouran A. Elattal, Amany L. Kansoh, Mona A. Esawy

**Affiliations:** 1 Chemistry of Natural and Microbial Products Department, Pharmaceutical Industries and Drug Research Institute, National Research Centre, Dokki, Cairo, 12622 Egypt; 2 Microbial Chemistry Department, National Research Centre, Dokki, Cairo Egypt

**Keywords:** *Bacillus amyloliquefacien*, Milk clotting, Cell immobilization, Thermodynamic-cheese

## Abstract

**Background:**

Milk clotting enzymes, essential for milk coagulation in cheese production, are obtained from the stomach of young ruminants, an expensive and limited source. This study was accomplished by finding a suitable alternative. Bacterial isolates recovered from honey were screened for milk clotting enzyme activity. and further, by immobilization of the microorganisms to enhance stability and facilitate their repeated use.

**Result:**

The most effective enzyme was produced by a microbe identified as *Bacillus amyloliquefaciens* based on 16 S rRNA sequencing. The cells were encapsulated in Ca^2+^ alginate beads. These beads retained complete enzyme production after being used five times. Glucose and Soybean were selected as the most favorable carbon and nitrogen sources, respectively. The optimum temperature for activity was 35 ℃ for both free and immobilized cells but as the temperature was increased to 55 °C and above, the encapsulated form retained more activity than the free cells. The pH optimum shifted from 6.5 to 7 for the free cells to 7–7.5 for the immobilized cells. The immobilization process decreased the activation energy for enzyme production and activity, prolonged the enzyme half-life, and increased the deactivation energy. Enzyme produced by immobilized cells generated a more compact cheese.

**Conclusions:**

The finding of this study was to identify a less expensive source of milk-clotting enzymes and confirm the success of cell immobilization in improving cell rigidity and stability. Also, immobilization of this *B. amyloliquefaciens* strain offers an enzyme source of value for industrial production of cheese.

## Introduction

Chymosin, the main component of rennet (milk clotting enzyme), is an acid protease produced in the fourth stomach of young, milk-fed ruminants, such as calves, lambs and kids. It serves as a milk coagulant in the making of cheese by hydrolyzing the kappa-casein chain in milk’s casein micelles [1, 2]. The type of milk-clotting enzyme (MCE), which is essential in the process of milk coagulation and cheese maturation, has a significant impact on the quality of the cheese. The high cost and limited availability of rennet from young animals, as well as its unsuitability for vegetarians, has led to numerous projects focused on finding other sources of MCE. A number of microorganisms have been identified as being particularly useful since they grow rapidly, are relatively inexpensive to produce and have a diversity of properties desired by cheese makers [3, 4]. Different species of Bacillus supply some of the most popular distinguishing traits. For instance, lipase from *Bacillus licheniformis* NCU CS-5 was used to enhance the fatty acid flavor release for low-fat cheeses [5]. *Bacillus velezensis* DB219, *Bacillus subtilis* PNG27, and *Bacillus methanolicus* LB-1 have been reported as milk clotting producers [6]; [7]; [3]. Several studies have used honey as a reservoir for potential probiotic and prebiotic bacteria [8–11].

*Bacillus amyloliquefaciens* is an efficient source of milk clotting enzymes [12]. Immobilization of microbial cells is considered one of the best methods to reduce the economic cost of enzyme production because the cells can be reused several times without losing too much enzyme activity. Moreover, cell immobilization improves enzyme stability, facilitates cell separation from the medium, enhances retention of plasmids, increases resistance to toxic contaminants, and promotes secondary metabolite production [13]. Calcium alginate beads are often used for cell immobilization. The beads offer superior biocompatibility, affordability, accessibility, and ease of preparation [14]. Thermodynamic studies help evaluate the suitability of an enzyme for a given application.

This study represents a new attempt to address the clotting problem that has hindered the application of milk clotting in the industrial field. To this end, *Bacillus amyloliquefaciens* cells were immobilized on Ca^2+^ alginate beads. The cells exhibited high stability and could be reused for 5 cycles with no loss of milk clotting enzyme production. Abiotic production parameters were optimized. A thermodynamic study confirmed that immobilization decreases the activation energy and increased the deactivation energy while prolonging the enzyme half-life. Immobilization of this *B. amyloliquefaciens* strain offers an enzyme source of value for industrial production of cheese.

## Materials and methods

### Bacterial sources

Different honey types were collected from Saudi Arabian Gably honey, Libyan Gably honey and commercial Egyptian honey made from nectar collected from clover.

#### Isolation of bacterial strains

One hundred microliters of each honey sample was spread on Nutrient agar medium (Peptone, 5.0; Beef extract, 3.0; NaCl, 3.0 and agar, 15.0 gm/L) [15]. The plates were incubated at 37 °C for 24 h or until the bacterial colonies were grown to sufficient size for colony replication (3–5 mm in diameter). Four bacterial colonies were selected and purified by serial subculture and plating. Selected bacteria were stored at − 80 °C in the culture medium with glycerol. The bacterial isolates were streaked onto agar slants and preserved at 4 °C.

### Screening isolates for milk clotting enzyme production

All pure isolates were inoculated on milk plates to test for milk clotting enzyme secretion and incubated at 37 °C for 28 h. Milk clotting positive strains were determined by the presence of a clear zone around the colony on the milk agar plates indicating milk hydrolysis. Colonies having a clear zone around them were selected for further investigation [16].

### Identification of potent milk clotting producers by DNA and 16 S- rRNA

Bacterial isolates were grown in Luria-Bertani broth (LB) for 24 h and were harvested by centrifugation at 12,000 g for 5 min. They were washed three times by resuspension in 0.85% NaCl and centrifugation. Genomic DNA was extracted using Gene JET Genomic DNA purification kit (Thermo Scientific, Lithuania) according to the manufacturer’s recommendations [17].

Amplification was done using forward primer 8 F (5′-CAG GCC TAA CAC ATG CAA GTC-3′) and reverse primer 1492R (5′-GGG CGG GGT GTACAA GGC-3′). The PCR mixture was carried out in a volume of 50 µl, containing 22 µl of MQ, 25 µl of DreamTaq Green DNA Polymerase (Thermo Fisher Scientific, USA), 1 µl of each forward and reverse primer (10 µmol/l), and 1 µl of template. The PCR amplification conditions were 4 min of preheating at 95 °C, 30 s denaturation at 95 ℃, 45 s of primer annealing at 50 ℃, 1 min extension step at 72 °C, and post cycling extension of 10 min at 72 ℃ for 35 cycles. The reactions were carried out in a thermal cycler (Applied Biosystem Thermal Cycler, USA) [18].

### Agarose gel preparation

Agarose was boiled in 1X TBE buffer. Ethidium bromide was added to the melted gel after the temperature cooled to 55 °C. Melted gel was poured into the mini-gel apparatus tray and the comb was immediately inserted. The comb was removed after the gel hardened. Electrophoreses buffer (1X TBE) covered the gel and 15 µl of dsDNA was loaded in each well and 3 µl of 2.5 kbp DNA ladder was added. 16 S rDNA PCR product was extracted from the gel using Promega Wizard Genomic DNA Purification Kit [19].

### Sequence alignment, phylogenetic analysis, and bioinformatics analysis

Amplified PCR product was purified and sequenced at Macrogen, Inc., Korea. Raw sequencing data was edited (contig and peak chromatogram verification) using the Finch T.V 1.4.0 program. Analysis of 16 s rRNA sequences of strains was performed using the BLAST (N) program of the National Center of Biotechnology Information (NCBI) (Rockville Pike, Bethesda MD, USA). Multiple sequence alignment was done using the Clustal W 2.1 program. The phylogenetic trees were constructed using the neighbor joining method by MEGA. X.

### Enzyme production

Milk clotting enzyme was produced according to Guleria et al. 2016 [12]. The medium used for MCE production had the following composition (g/L): Glucose 10, yeast extract 2.5, Casein 5. The pH was adjusted to 7.0 prior to sterilization. One ml of cell suspension from a 24 h-old slant was transferred to 50 ml sterile medium in a 250-ml Erlenmeyer ask. The flasks were incubated at 35 °C on a rotary shaker at 180 rpm for 24 h. After incubation, the medium was centrifuged at 6000 x g and 4 °C for 10 min and the cell free filtrate was considered as the source of crude enzyme.

### Milk clotting activity

Milk-clotting activity (MCA) was determined according to the method of Arima et al. [20] and expressed in terms of Soxhlet units (SU). One SU was defined as the amount of enzyme which clotted 1 ml of a solution containing 0.1 g skim milk powder and 0.00111 g calcium chloride in 40 min at 35 °C. In brief, 0.5 ml of material was added to a test tube containing 5 ml of reconstituted skim milk solution (10 g dry skim milk/100 ml, 0.01 M CaCl2) pre-incubated at 35 °C for 5 min. The solution was mixed well, and the clotting time T (s), measured as the time period from the addition of test material to the first appearance of clots, was recorded and the clotting activity was calculated using the following formula: Soxhlet units (SU) = 2400 × 5 × D/T × 0.5; T = clotting time (s); D = Dilution of test material [16].

### Protein determination

The method used to measure protein content was disclosed by Lowry et al. [21]. By deducting the amount of unbound protein from the protein that was initially introduced for immobilization, the amount of protein that was immobilized could be estimated.

### Protease activity (PA)

To determine proteolytic activity, the bacterial dilutions were streaked on milk agar plates. The plates were incubated for 24 h at 30 ℃, and strains that produced clear zones in the medium were chosen for additional testing. The quantitative protease activity was measured according to Kembhavi & Kulkami [22]. One unit of protease activity was the amount of enzyme that released one micromole of equivalent L-tyrosine/min under the standard conditions of the assay.

### Microencapsulation of bacterial cells in Alginate beads

For microencapsulation of bacterial cells, all solutions and equipment were sterilized at 121 °C for 20 min. Alginate beads were prepared according to Bashan [23]. In brief, 250 mL of bacterial culture was centrifuged, the supernatant was removed, and the cell pellet was washed with saline solution. It was then suspended in 50 mL of a 3% alginate solution and thoroughly mixed. This mixture (alginate and cell pellet) was dropped into a sterile 3% calcium chloride solution using a syringe needle, with 10 cm between the needle and the calcium chloride solution surface. The alginate beads containing bacterial cells were allowed to harden in a calcium chloride solution for 3 h. Beads were collected, washed with sterile water, and stored in saline solution for further investigation [24].

### Operational stability of immobilized cells

Operational stability was determined using 1 g of cells immobilized in calcium alginate beads. The immobilized form was incubated with 50 ml of the medium in a 250 ml flask for 24 h at 180 rpm and 35 °C. Then it was collected, washed with distilled water, and re-suspended in 50 ml of freshly prepared medium to start a new run. The supernatant was assayed for milk clotting activity after centrifugation. The amount of enzyme that leaked out of the beads was determined by dividing the optical density (OD) of the culture filtrate of the immobilized cells by the OD of the free cells and subtracting the obtained value from 100.

### Effect of incubation time and temperature on enzyme production

To study the effect of incubation time on the production of milk clotting enzymes from free and immobilized cells, the BM cultures were incubated for 24 h at 180 rpm and 37 ℃ for different times (6, 12, 24, 36, 48, 50, and 62 h). Then the enzyme production is determined according to the enzyme activity.”

The effect of temperature on free and immobilized cells was measured by preheating the free and immobilized cells at different temperatures (25–60 ℃) for different time intervals (15,30, 60,90 and 120 min). Then the preheated cells were inoculated in 50 ml of the production medium for 24 h at shaker (180 rpm, 40 ℃). The residual activity of enzyme production was assayed under optimized conditions. The optimum temperature was taken as 100% activity for complete enzyme production and the relative activity of the produced enzyme at each temperature was expressed as a percentage related to the 100% activity.

The activation energy (Ea) was calculated from the slope of the Arrhenius plot [25] of 1000/T versus ln [enzyme relative activity] (Ea = − slope × R), where R (Gas constant) = 8.314 J. K. mol^− 1^.

### Effect of carbon and nitrogen sources on enzyme production

To evaluate the most appropriate carbon source for production of milk clotting enzyme the glucose in the medium was replaced with 1% (w/v) of different carbon sources (fructose, lactose, mannose, maltose, sucrose, cellulose, and starch). Free and immobilized cultures were incubated at 35 °C for 24 h. Then milk clotting enzyme activity was determined.

The medium was supplemented with equimolar amounts of different organic and inorganic nitrogen (casein, peptone, urea, soyabean, NH_4_Cl, KNO_3_, NaNO_3_) and both free and immobilized cultures were incubated at 35 °C for 24 h. Then milk clotting activity was determined.

### Effect of temperature on enzyme activity

The optimal temperature was determined by incubating the reaction mixture at different temperatures from 25 to 60 °C. The residual activity of enzyme was assayed under optimized conditions. The optimum temperature was taken as 100% activity the relative activity at each temperature was expressed as a percentage related to the 100% activity.

### Effect of pH on the production of milk clotting enzyme

The cell culture for both free and immobilized cells were adjusted at pHs ranging from (5–9) in acetate buffer for pH 5–6 or in phosphate buffer for pH 7–9. The BM cultures were incubated at180 rpm and 35 °C for 24 h and then assayed for milk clotting production.

### Temperature stability

The effect of temperature on free and immobilized cells was measured by preheating the free and immobilized cells at different temperatures (25–60 ℃) for different time intervals (15,30, 45 and 60 min). Then the preheated cells were inoculated in 50 ml of the production medium for 24 h at shaker (180 rpm, 35 ℃). The residual activity was assayed under optimized conditions. The optimum temperature was taken as 100% activity of enzyme production and the relative activity at each temperature was expressed as a percentage related to the 100% activity.

### Thermodynamic study

#### Thermal inactivation

The dissociation constant (kd) was estimated by a regression plot of relative activity (log) versus time (min). The half-life (T_1/2_) for the enzyme was determined by T_1/2_=ln2/kd. The decimal reduction time or the time needed to reduce the enzyme value by 90% was calculated as D-value = ln10/kd.

The activation energy (Ea) of catalysis for both the free and immobilized milk clotting enzyme forms was determined from the slope of the Arrhenius plot [log V (ln of % residual activity) versus reciprocal of absolute temperature in Kelvin (1000/T)], which is calculated as Slope = − Ea /R where R is the gas constant (8.314 mol − 1 k − 1). The denaturation energy for the enzyme was determined from a plot of the log of the denaturation rate constants (ln kd) versus the reciprocal of the absolute temperature (K) as follows: Slope = Ed/R.

### pH stability

The effect of pH on free and immobilized cells was measured by subjecting the free and immobilized cells to pHs ranging from 5 to 9 for time intervals of 15, 30, 45, and 60 min. Then the treated cells were inoculated in 50 mL of the production medium for 24 h at 150 rpm, 35ºC, and the residual activity was assayed under optimized conditions. The optimum pH was taken as 100% activity of enzyme production, and the relative activity at each pH was expressed as a percentage of the 100% activity.

### Effect of metal ions on production of the milk clotting enzyme

Some metal ions (MgSO_4_.7H_2_O, NaCl, FeSO_4_, CaCl_2_, ZnSO_4_, MnSO_4_ and CuSO_4_) were added separately to the culture medium at a final concentration of 0.01 M and then inoculated by free and immobilized cells incubated at 35 ºC for (24 h free cells, 48 h immobilized cells). The enzyme production was determined as before.

### Statistical analysis

Data analysis was carried out with MICROSOFT EXCEL (2007). All data are presented as means ± standard error of means. The acid and alkali tolerance experiments, the bile and pancreatic enzyme tolerance were independently replicated 2 times (*n* = 2), with 2 measurements per replicate. The mean of the repeated measurements yielded the value for each replicate.

## Result and discussion

### Isolation and identification

A preliminary screening of 24 honey isolates revealed variations in the size of the inhibition zones produced on skim milk agar plates, indicative of milk clotting activity. Isolate NO 2 showed the largest inhibition zone and the highest enzyme activity (Table 1). Of 24 isolates only 14 isolates gave a positive result.

**Table 1:** Screening for milk clotting production

Number of strain	Diameter of clear zone (Mm)	Activity of enzyme (SU/ml)
1	-	-
2	25	2000 ± 086
3	-	-
4	14	600 ± 066
5	13	400 ± 055
6	12	343 ± 076
7	13	218 ± 006
8	-	-
9	-	-
10	-	-
11	20	1200 ± 086
12	12	240 ± 086
13	-	-
14	32	2400 ± 006
15	11	110 ± 077
16	17	750 ± 055
17	12	480 ± 049
18	15	800 ± 090
19	13	600 ± 048
20	18	1200 ± 044
21	-	-
22	-	-
23	-	-
24	-	-

**Fig. 1:**
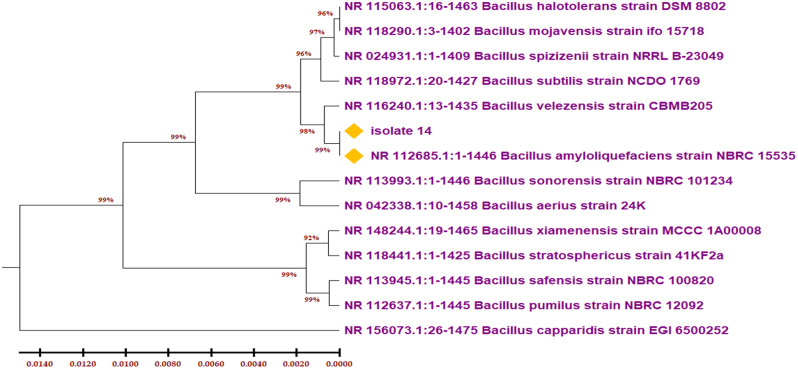
Phylogenetic tree based on partial 16 S rDNA sequences, showing the relationship between isolate No (14) *Bacillus amyloliquefaciens* and other species belong to the genus bacillus. The tree was constructed using the MEGA11 and neighbor-joining method

The isolate was identified as *Bacillus amyloliquefaciens* based on 16 S rRNA sequencing. The phylogenetic tree of the isolate (Fig. 1) shows that it belongs to the genus Bacillus and is 99.93% homologous to *B. amyloliquefaciens* strain NBRC15535.

Many authors have reported *B. amyloliquefaciens* as an efficient producer of milk clotting enzymes. For example, Zhao et al. [26] detected a novel milk-clotting enzyme produced by *B. amyloliquefaciens* GSBa-1. Similarly, Guleria et al. 2016 [12] isolated a milk-clotting enzyme-producing *B. amyloliquefaciens* from an apple rhizosphere in the northern Himalayas. Also, He et al. [27], mentioned *B. amyloliquefaciens* as a milk clotting enzyme producer from the Tibetan Plateau. Additionally, honey was previously mentioned as an efficient reservoir for probiotic bacteria with unique properties [28, 29]. These characteristics made *B. amyloliquefaciens* a promising candidate for use in the cheese industry [30].

### Cell immobilization and effect on reusability

In this study ***Bacillus amyloliquefaciens*** GSBa-1 cells were immobilized in Ca^+ 2^ alginate beads. As reported by many others working with various enzymes, milk clotting enzyme activity was higher when the enzyme was immobilized compared with the free enzyme perhaps because the enzyme is protected from harsh environmental conditions. In addition, the alginate matrix has additional advantages of being less expensive, nontoxic, and requiring mild conditions for biocatalyst synthesis [31].

**Fig. 2:**
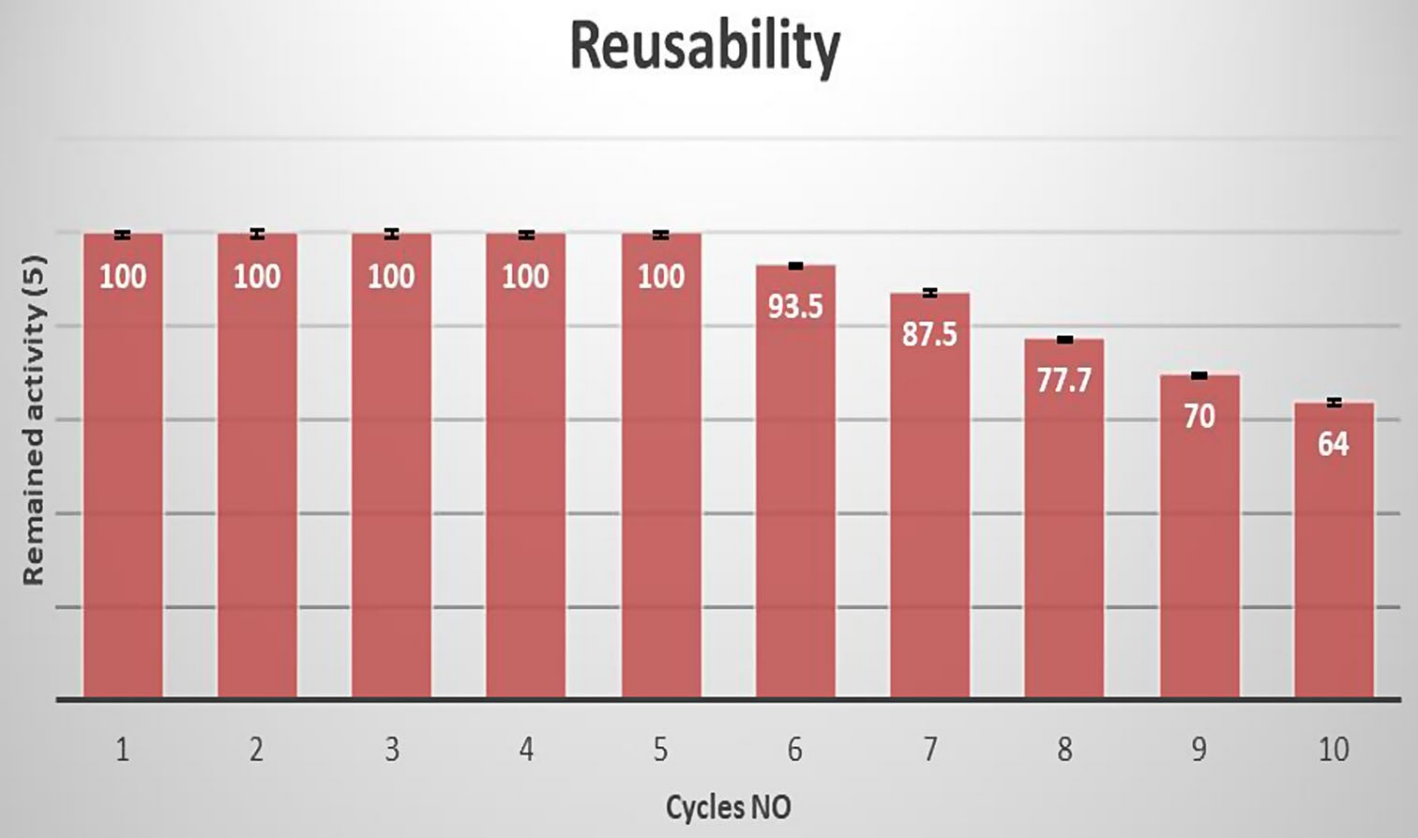
Reusability of the immobilized *Bacillus amyloliquefaciens* MCA

The main problem for MCE reusability is the coagulation around the carrier which may result in blocking the active site of the enzyme [32, 33]. In this study, it was determined that immobilized cells could be reused five times with no loss in activity. Complete milk clotting decreased gradually to 67% of the initial enzyme production after 10 cycles (Fig. 2). This suggests that the immobilization process improves the stability essential for cheese manufacture. Data from this study indicates immobilized *Bacillus amyloliquefaciens* produced stable and reusable MCE. Alginate is made up of β-d-mannuronate and α-l-guluronate residues, which are [1→4], linked. The gradual decrease in production after 5 cycles could possibly be the result of Ca^2+^ attaching to various α-guluronate residues, creating a gel network. Future work will study the possibility of inhibition of the gel network with monovalent metal ions, to extend the useful lifetime of the beads [34].

### Factors production of milk clotting enzyme

#### Incubation time and temperature, sources of carbon and nitrogen

The optimal time for milk clotting production (Table 1) was obtained after 24 h and 36 h. Below and above this time slot there was a reduction in enzyme production. Similar results were reported by Guleria et al. [12], where the highest production of the *Bacillus amyloliquefaciens* SP1 milk clotting enzyme activity was obtained after 24 h. The optimum temperature for the immobilized and free MCA production was 35 °C. Below and above this temperature there was a noticeable drop in MCA production (Table 1).

Lactose and glucose were the most effective carbon sources for milk-clotting enzyme production, with no noticeable difference between the two (Table 1.). Most proteolytic enzymes cause milk to coagulate, but because the coagulum is continuously digested by proteolysis, stable cheese is rarely produced. The optimal carbon source should be selected based on the desired proteolytic activity (PA). For cheese production, MCE with a high milk clotting activity to protease activity ratio is desired. In this study, the free enzyme had a lower PA in the presence of glucose than in the presence of lactose, with MCA to PA ratios of 9600 and 2086, respectively. The immobilized enzyme also had a lower PA in the presence of glucose than in the presence of lactose, with MCA to PA ratios of 9600 and 2307, respectively.

Other tested carbon sources had an adverse effect on MCE production, especially cellulose and starch. Starch can activate amylase production and cellulose can result in the production of cellulases. Under these circumstances, milk clotting production will be reduced. Shieh et al. [35], reported that glucose followed by sucrose were the most effective carbon sources for *Bacillus subtilis* natto MCE production. However, our results showed that lactose inhibited enzyme activity completely.

Soyabean and peptone were the most effective nitrogen sources for MCE production. The protease activity of the free enzyme was 0.272 and 0.26 mg/mL, with MCA to PA ratios of 11,029 and 9230, respectively. The immobilized enzyme had a PA of 0.65 and 0.51 mg/g, with MCA to PA ratios of 5882 and 4615, respectively. Therefore, Soybean was selected as the best nitrogen source under the selected conditions.

### Optimum temperature for enzyme activity

**Fig. 3:**
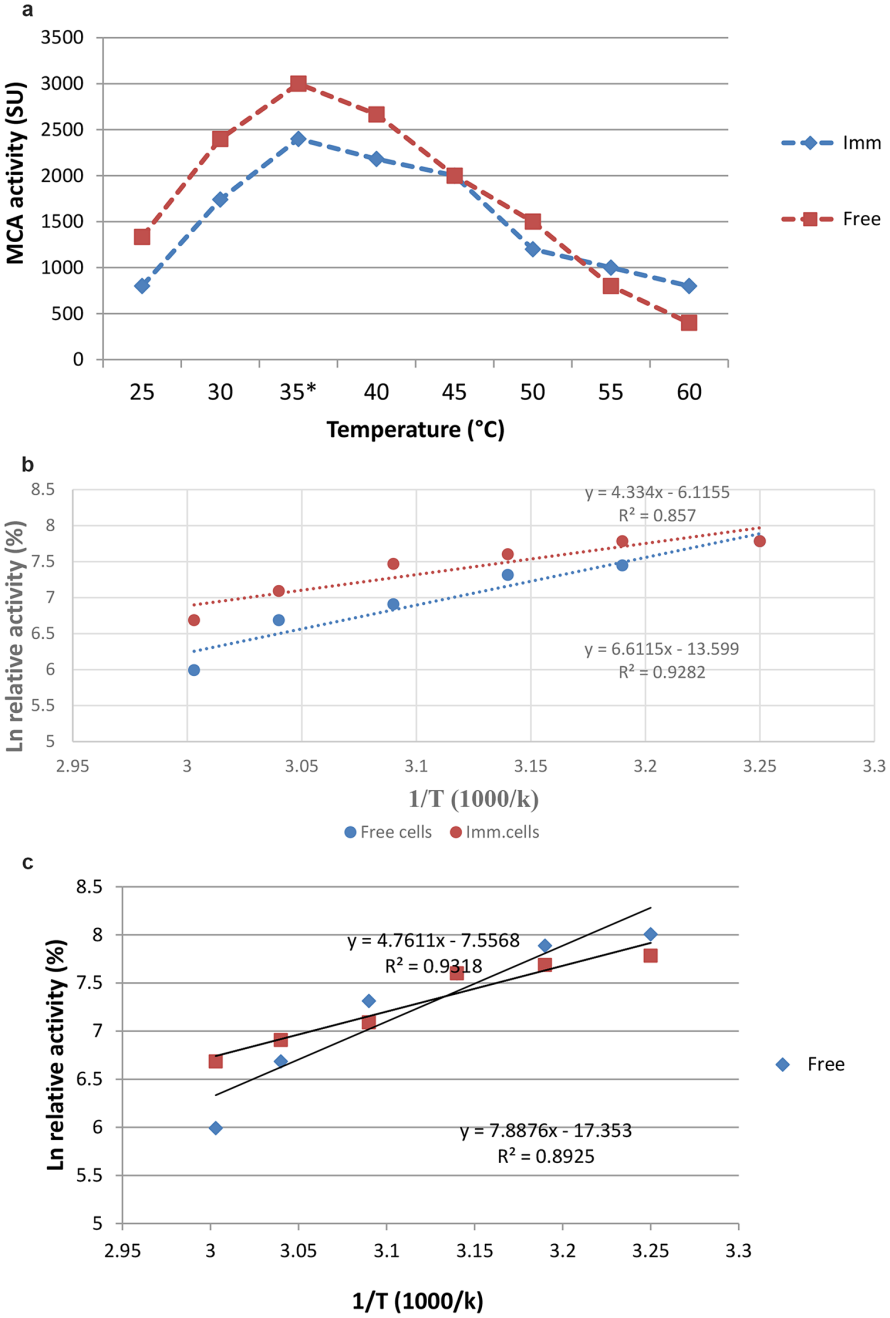
Determination the optimum temperature for both the free and immobilized *Bacillus amyloliquefaciens* milk clotting **(a)**, Ea for enzyme production **(b)**, Ea for enzyme activity **(c)**

The optimum temperature for the enzyme obtained from the free and immobilized cells was 35 ℃ (Fig. 3a). Also, the results showed that the activity from the immobilized enzyme was higher as the temperature increased in comparison to the free enzyme. This result is further evidence that cell immobilization tends to protect cells against environmental conditions and gave the enzyme rigidity.

#### Evaluation of the activation energy for enzyme production and activity

Additionally, immobilization reduced the activation energy (Ea) from 54.96 to 35.99 7 kJ mol^− 1^ for the enzyme production and from 64.20 to 38.75 7 kJ mol^− 1^ for the enzyme activity (Fig. 3. b, c) and accordingly, saving energy costs [36]. Wehaidy et al. [37, 38, 39] claimed the calculated Ea value of Ch-MCE was 1.4-fold lower than that of the free MCE, indicating that immobilization increased the enzyme’s catalytic efficiency by reducing the energy needed to form the activated complex of enzyme and substrate.

#### Optimum pH for enzyme production

**Fig. 4:**
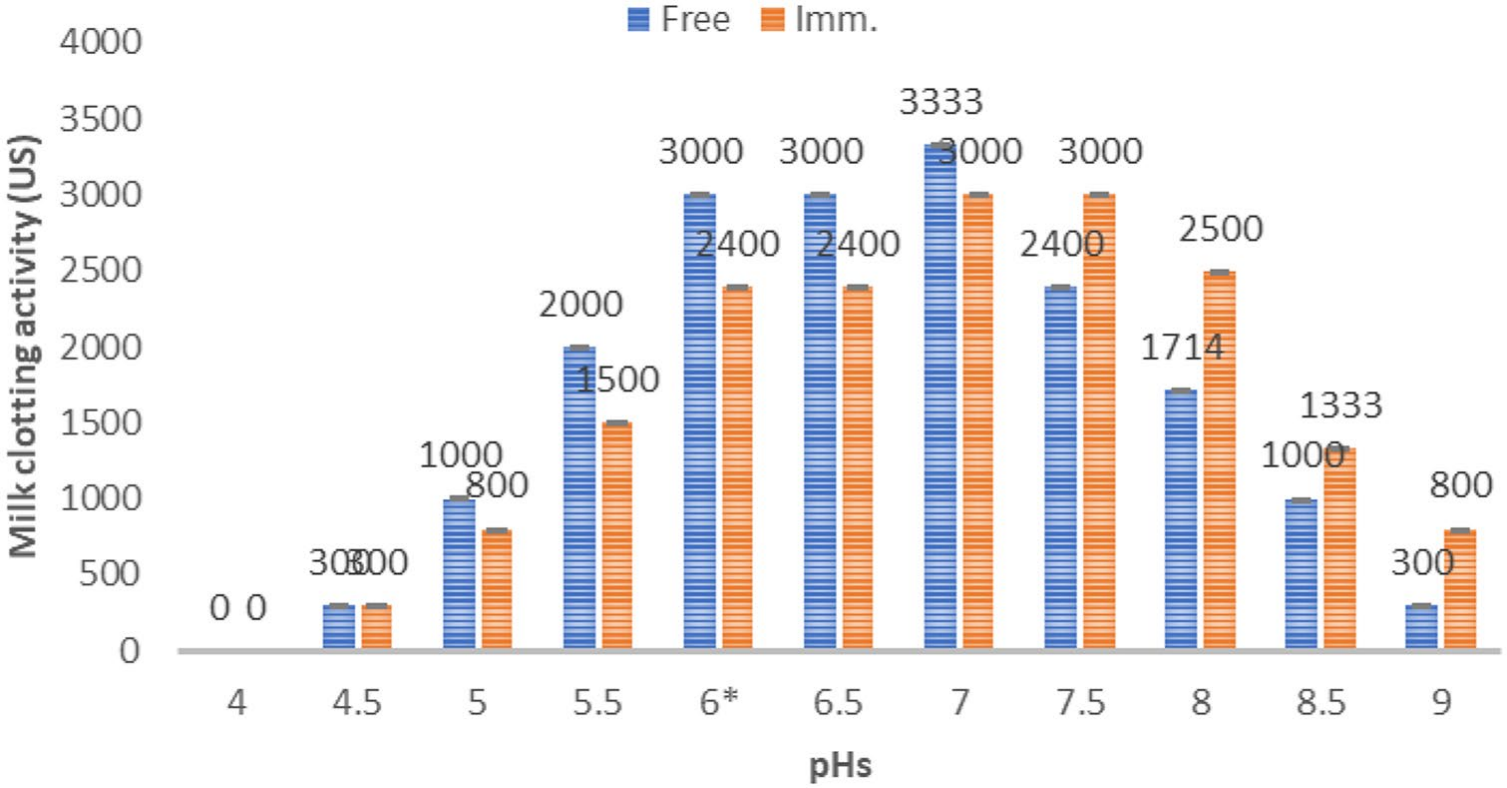
Determination the optimum pHs for both the free and immobilized *Bacillus amyloliquefaciens* milk clotting

The pH has an important role in milk clotting activity (Fig. 4.). The maximum enzyme activity was pH 7 for the free enzyme and 7-7.5 for the enzyme obtained from the immobilized cells. A similar result was obtained by Esawy & Blanc [32, 33] who mentioned that the optimum activity of free and immobilized *Bacillus licheniformis* 5A1 MCA was obtained at pH 7. This result indicated that the cell immobilization process protected the cells from different environmental factors affecting the cell vulnerabilities.

#### Thermal stability

**Fig. 5:**
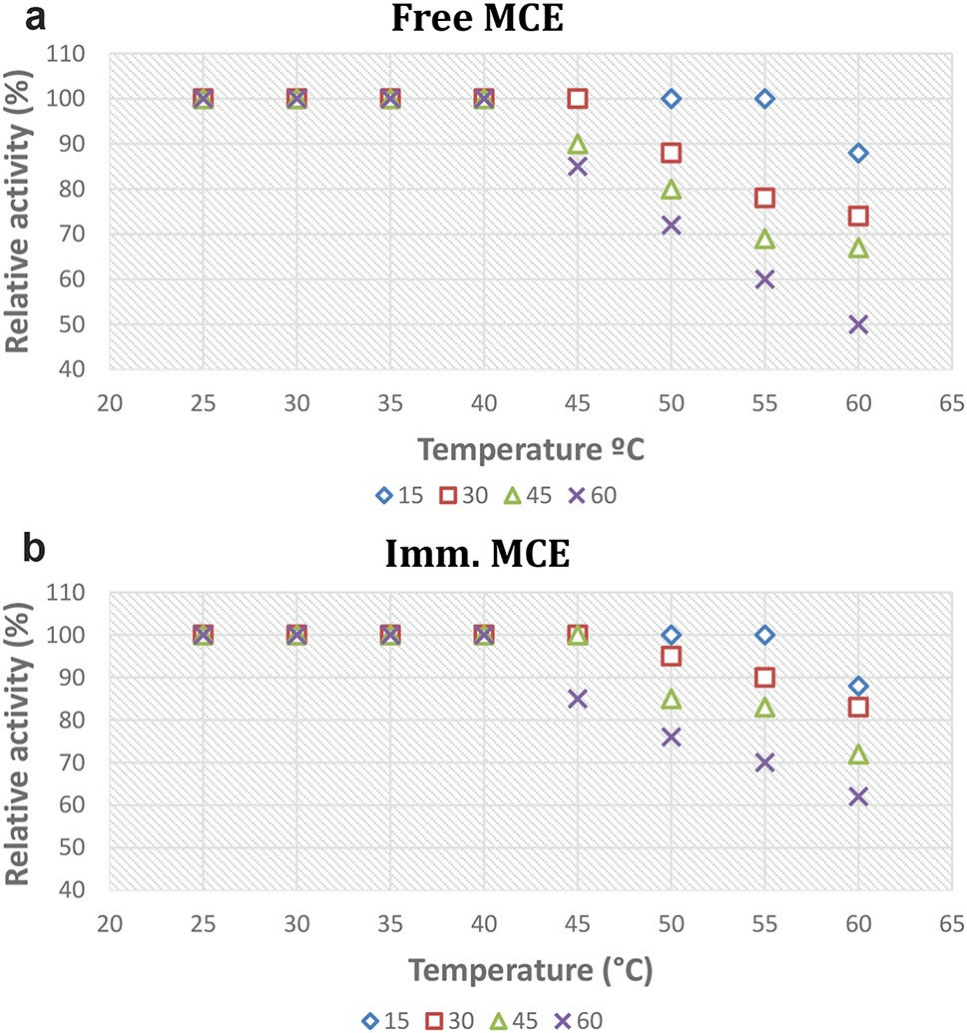
Thermal stability for the free MCA **(a)** and the immobilized MCA **(b)**

One of the most critical application requirements was the temperature stability of the immobilized cells. The free and immobilized milk clotting producer cells had 100% activity below 40 °C. Starting from 45 °C immobilized cells enzyme was more tolerant to denaturation than free cells as the incubation time increased. For instance, at 60 °C and one h incubation the free form lost 43% of its original production while the immobilized form lost 33% of its original MCE production (Fig. 5a, b). This result demonstrated the protective effect of alginate immobilization on cell hardness, which acquired enzyme stability and tolerance to the severe conditions. Milk clotting enzyme was incubated for 15 min at temperatures ranging from 30 to 60 °C. With a peak activity at 30 °C, the relative milk clotting activity decreased as the temperature rose but as the temperature reach 65 °C, the enzyme became unstable and milk clotting activity was completely lost [1].

### Thermodynamic study

**Fig. 6:**
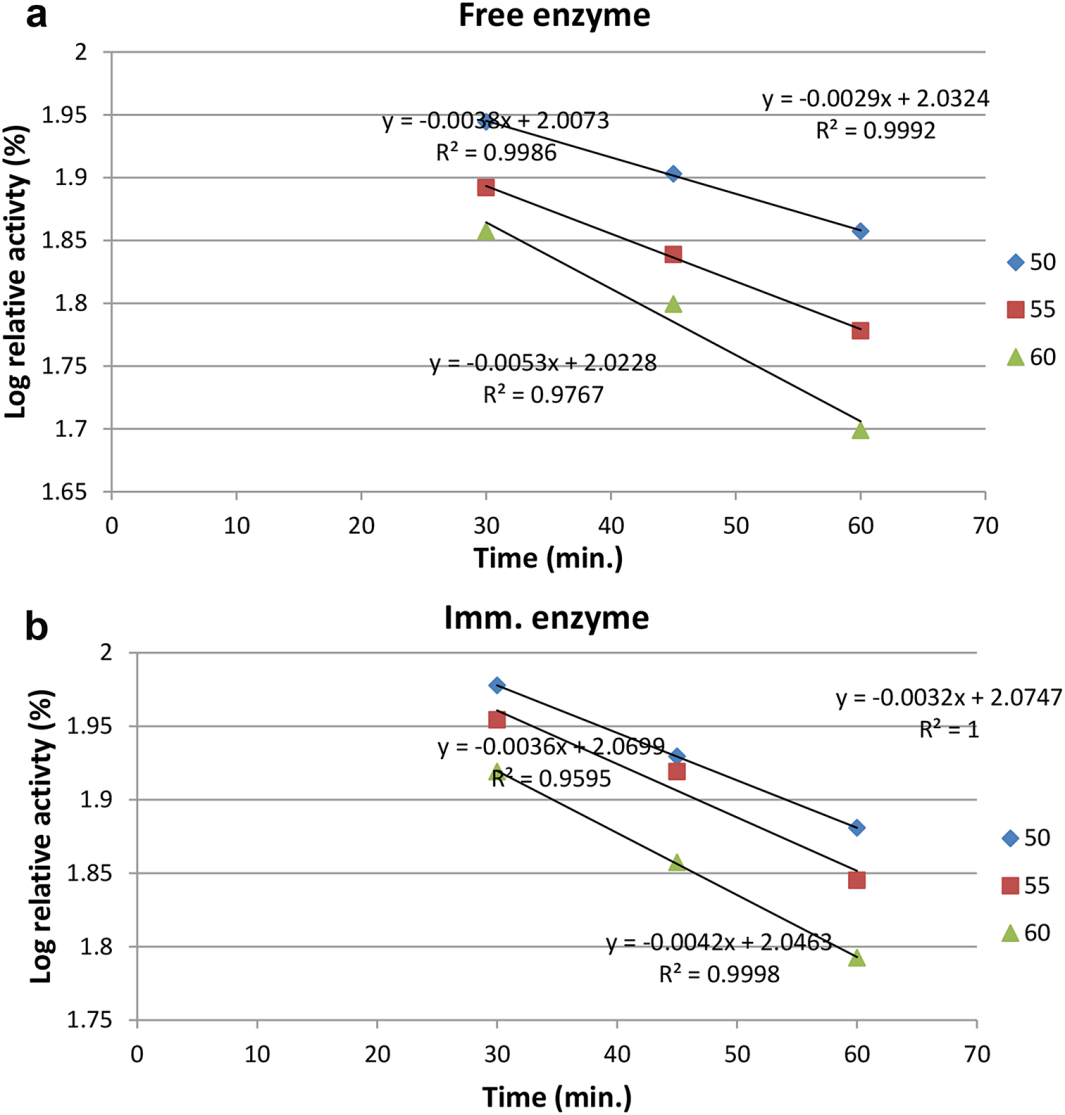
Deactivation rate constant for the free MCA **(a)** and the immobilized MCA **(b)**

**Fig. 7:**
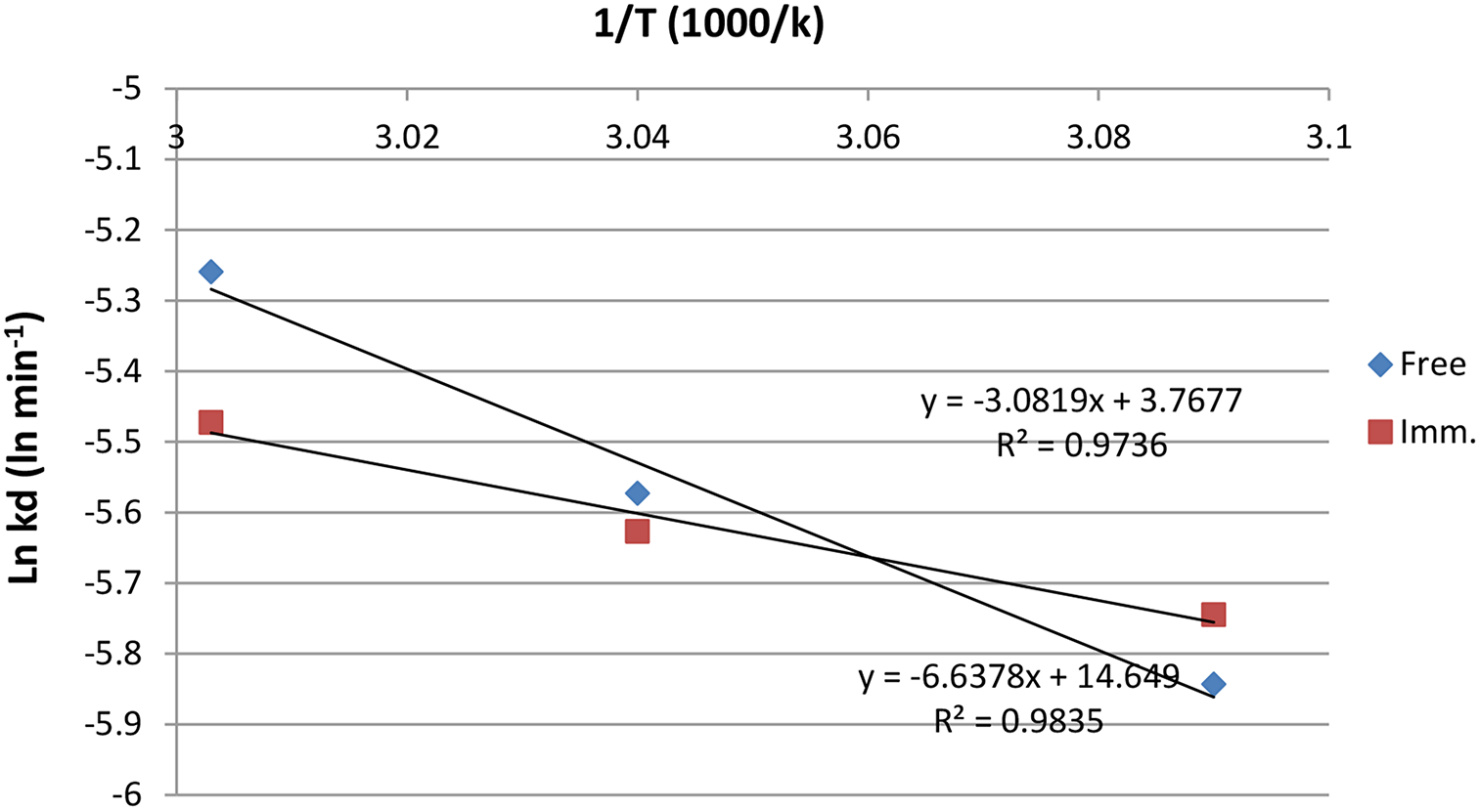
Arrhenius plot to calculate activation energy for denaturation (Ed) for the free and immobilized MCA

The deactivation rate constant was determined as shown in Fig. 6a, b for the *Bacillus amyloliquefacien* milk clotting produced from the free and immobilized cells, aiming to evaluate Ed, t_1/2_, and D values. These parameters gave a clear picture of the extent to which cell immobilization succeeded. The data demonstrated that first-order kinetics was present in the linear log relative activity versus time plots. Also, the determination of the denaturation energy (Ed) demonstrated the vital role of cells immobilization in enzyme stability, where the deactivation energy of the milk clotting produced from the immobilized cells increased about 2-fold (from 25.62 kJ mol − 1 to 55.19 kJ mol − 1 (Fig. 7) compared with the free cells. This indicates that the immobilized enzyme required additional energy to denature, which was a positive indication of an improvement in thermal stability [40]. A low Ed hinders application of the enzyme in the industrial field [41]. The t_1/2_ of the free and immobilized forms were determined at 50, 55, 60 ℃. The t_1/2_ of the enzyme produced from the free cells was 238, 182, 133, minutes and immobilized enzyme was 216, 192, and 164 min respectively. The D values for free enzyme were 793, 605, and 442 min. For enzyme produced from immobilized cells, the D values were 718, 638, and 547 min. The previous results indicate the success of the cell immobilization process in increasing the cell’s rigidity and stability, allowing the immobilized cells to be reused several times without enzyme loss.

### pH stability

The free and immobilized cells was incubated at pHs 5–9 for different time intervals. Both the free and immobilized forms showed complete stability up to one hour at pHs 5–7. In industrial applications, there is a pressing need for pH stability in this range [42]. At pH 8, the enzyme produced from the free cells gradually lost up to 73% of its original production after one hour. On the other hand, the enzyme produced from the immobilized cells kept its full production for 45 min at pH 8 and only lost 5% of its original production after one hour. At pH 9, the role of the cells immobilization process in enzyme protection was clearer, since the free enzyme lost 35% of its original production while the immobilized enzyme lost only 15% after one hour (Table 2). This result concluded that the cells tolerance and stability increased after the cell immobilization. Since it shielded the cells from toxic materials and harsh environments caused by pH extremes and accordingly protected the produced enzyme to some extent. According to Pervez et al. [43], immobilization reduces the inhibition of enzymes either by stabilizing the structure of the enzyme or by removing the inhibitor.

**Table 2:** Effect of different parameters on milk clotting production

Incubation time (h)	6	12	24	36	48	50	62	-
Activity (US) Free enzyme	400 ± 026	1200 ± 022	2400 ± 043	2400 ± 023	1750 ± 055	1000 ± 065	300 ± 045	-
Activity (US) Imm enzyme	300 ± 007	1000 ± 065	2400 ± 046	2000 ± 096	1750 ± 034	1200 ± 081	800 ± 082	-
Temperature °C	25	30	35	40	45	50	55	60
Activity (US) Free enzyme	400 ± 086	1200 ± 086	2400 ± 086	2400 ± 086	2000 ± 086	1750 ± 086	1200 ± 086	800 ± 086
Activity (US) Imm enzyme	300 ± 086	1500 ± 010	2400 ± 20	1714 ± 090	1500 ± 20	1000 ± 099	800 ± 076	400 ± 086
Carbon sources (1%)	Lactose	Glucose	Fructose	Mannose	Maltose	Sucrose	Starch	Cellulose
Activity (US) Free enzyme	2400 ± 190	2400 ± 310	2000 ± 086	2181 ± 097	2000 ± 099	2000 ± 200	1714 ± 180	1500 ± 190
Activity (US) Imm enzyme	2400 ± 20	2400 ± 099	1714 ± 190	1500 ± 200	2181 ± 240	2000 ± 220	1500 ± 190	1333 ± 086
Nitrogen source (equimolar amount)	peptone	Casein (control)	Gelatin	Na NO_3_	Urea	Soya been	KNO_3_	Na NO_3_
Activity (US) Free enzyme	3000 ± 099	2400 ± 088	2181 ± 099	800 ± 190	1741 ± 160	3000 ± 320	1500 ± 086	2000 ± 086
Activity (US) Imm enzyme	2500 ± 220	2400 ± 080	1500 ± 155	800 ± 086	1714 ± 077	3000 ± 090	1500 ± 186	2000 ± 069

**Figs. 8:**
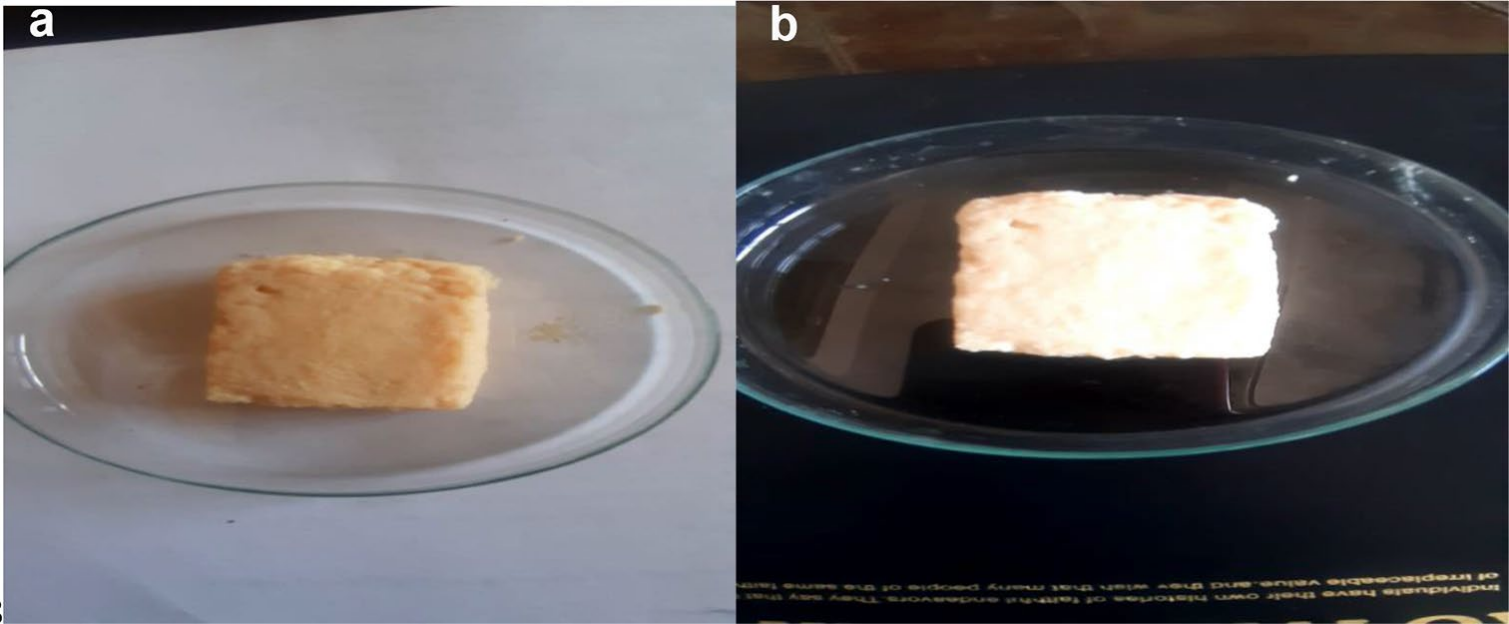
The picture of the cheese formed by the CF of the free **(a)** and immobilized enzymes **(b)** at the optimum conditions for enzyme activity

Figure 8a, b show examples of cheese formation using the culture filtrate of the free and immobilized cells (Fig. 8b) under the optimum conditions for enzyme activity. It was noticed that the cheese was more compact when using the culture filtrate of the immobilized cells compared with the culture filtrate of the free cells. This could be due to the fact that the milk clotting resulting from the immobilized cells was more efficient than the free form. Further study will be done to check the cheese texture, taste, and quality.

### Effect of different metal ions

As seen in Table 4, all tested metals had adverse effects on MCE production on both forms except NaCl and MnSO_4_. It was shown that NaCl and MnSO_4_ increased the production of the free forms by about 15.8% and 9.4% respectively. For immobilized enzyme, the metals increased enzyme production by 19.9% and 19.3% correspondingly. The activation of enzyme production by NaCl could reflect the fact that the microorganisms were collected from honey, an osmophilic medium [44]. Further, cell immobilization shortens the non-productive development phase, it enables more efficient operation. It has been suggested that immobilized cells with high cell densities increase product yield [45]. Pushkarev et al. [46] demonstrated that the milk-clotting activity of a mutant version of recombinant reindeer (Rangifer tarandus) chymosin was stimulated by Mg^2+^, Mn^2+^, and Ca^2+^. The result deduced that *Bacillus amyloliquefaciens* milk clotting was Ca^2+^-independent.

**Table 3:** Effect of pH stability on the activity of milk clotting enzyme for free and immobilized cell

pH	Milk clotting activity (MCA)(%)							
	Free cell				Immobilized cell			
Time	15 min	30 min	45 min	60 min	15 min	30 min	45 min	60 min
5	100 ± 006	100 ± 003	100 ± 055	100 ± 009	100 ± 007	100 ± 086	100 ± 001	100 ± 000
6	100 ± 000	100 ± 006	100 ± 003	100 ± 000	100 ± 001	100 ± 001	100 ± 002	100 ± 001
7	100 ± 086	100 ± 066	100 ± 086	100 ± 086	100 ± 086	100 ± 086	100 ± 086	100 ± 086
8	100 ± 088	95 ± 056	86 ± 066	73 ± 007	100 ± 086	100 ± 086	100 ± 086	95 ± 086
9	100 ± 004	86 ± 006	70 ± 080	65 ± 056	100 ± 022	100 ± 001	90 ± 006	86 ± 005

**Table 4:**
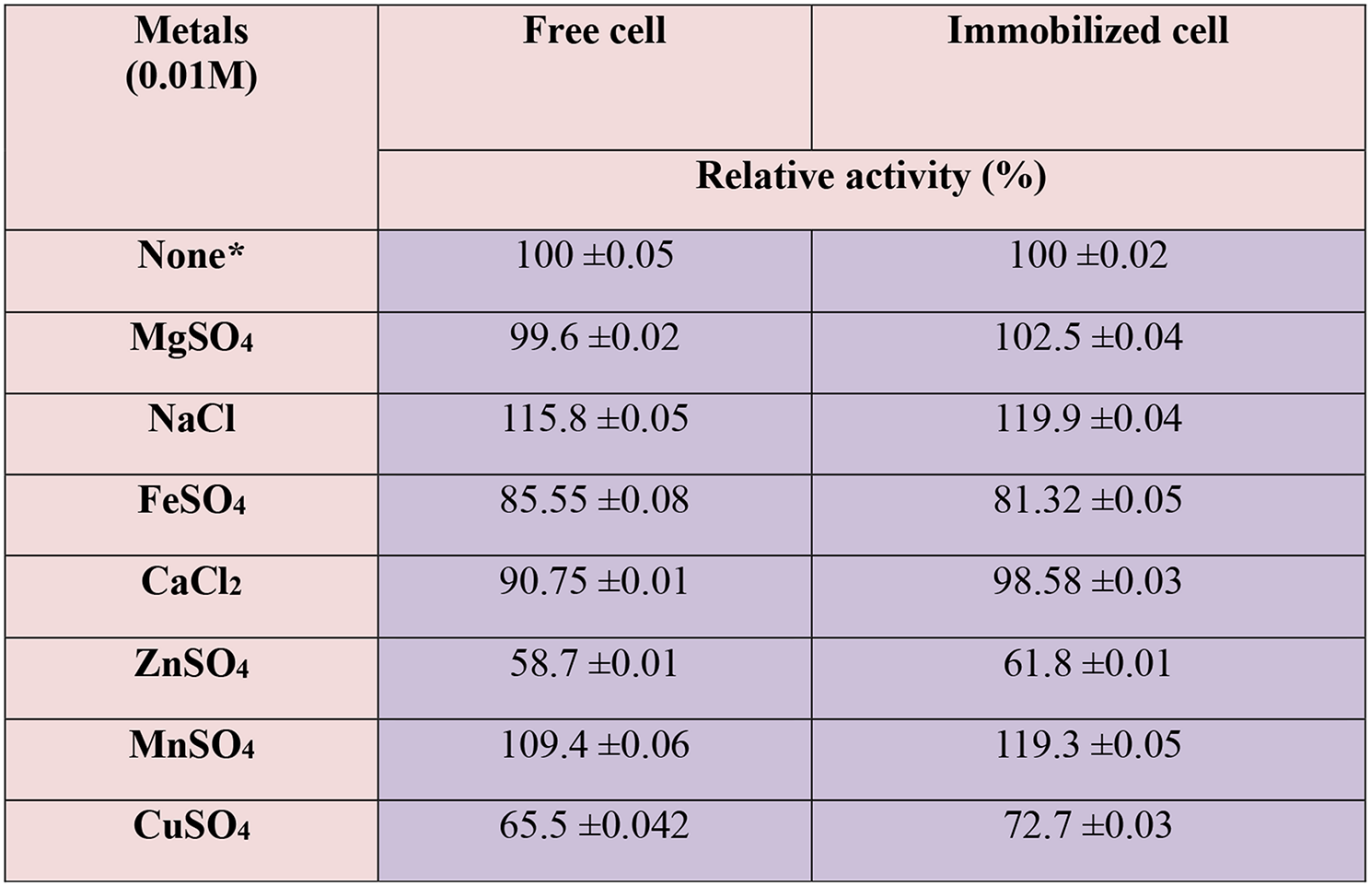
Effect of different metal ions on *Bacillus amyloliquefaciens* milk clotting

## Data Availability

The authors confirm that the data supporting the findings of this study are available within the article.
